# Comprehensive benchmarking of Markov chain Monte Carlo methods for dynamical systems

**DOI:** 10.1186/s12918-017-0433-1

**Published:** 2017-06-24

**Authors:** Benjamin Ballnus, Sabine Hug, Kathrin Hatz, Linus Görlitz, Jan Hasenauer, Fabian J. Theis

**Affiliations:** 1 Helmholtz Zentrum München - German Research Center for Environmental Health, Institute of Computational Biology, Ingolstädter Landstraße 1, Neuherberg, 85764 Germany; 2 Technische Universität München, Center for Mathematics, Chair of Mathematical Modeling of Biological Systems, Boltzmannstraße 15, Garching, 85748 Germany; 3 Bayer AG, Engineering & Technologies, Applied Mathematics, Kaiser-Wilhelm-Allee, Leverkusen, 51368 Germany

**Keywords:** Parameter estimation, Markov chain Monte Carlo, Sampling analysis, Benchmark collection, Ordinary differential equation, Systems biology

## Abstract

**Background:**

In quantitative biology, mathematical models are used to describe and analyze biological processes. The parameters of these models are usually unknown and need to be estimated from experimental data using statistical methods. In particular, Markov chain Monte Carlo (MCMC) methods have become increasingly popular as they allow for a rigorous analysis of parameter and prediction uncertainties without the need for assuming parameter identifiability or removing non-identifiable parameters. A broad spectrum of MCMC algorithms have been proposed, including single- and multi-chain approaches. However, selecting and tuning sampling algorithms suited for a given problem remains challenging and a comprehensive comparison of different methods is so far not available.

**Results:**

We present the results of a thorough benchmarking of state-of-the-art single- and multi-chain sampling methods, including Adaptive Metropolis, Delayed Rejection Adaptive Metropolis, Metropolis adjusted Langevin algorithm, Parallel Tempering and Parallel Hierarchical Sampling. Different initialization and adaptation schemes are considered. To ensure a comprehensive and fair comparison, we consider problems with a range of features such as bifurcations, periodical orbits, multistability of steady-state solutions and chaotic regimes. These problem properties give rise to various posterior distributions including uni- and multi-modal distributions and non-normally distributed mode tails. For an objective comparison, we developed a pipeline for the semi-automatic comparison of sampling results.

**Conclusion:**

The comparison of MCMC algorithms, initialization and adaptation schemes revealed that overall multi-chain algorithms perform better than single-chain algorithms. In some cases this performance can be further increased by using a preceding multi-start local optimization scheme. These results can inform the selection of sampling methods and the benchmark collection can serve for the evaluation of new algorithms. Furthermore, our results confirm the need to address exploration quality of MCMC chains before applying the commonly used quality measure of effective sample size to prevent false analysis conclusions.

**Electronic supplementary material:**

The online version of this article (doi:10.1186/s12918-017-0433-1) contains supplementary material, which is available to authorized users.

## Background

In the field of computational systems biology, mechanistic models are developed to explain experimental data, to gain a quantitative understanding of processes and to predict the process dynamics under new experimental conditions [1–3]. The parameters of these mechanistic models are typically unknown and need to be estimated from available experimental data. The parameter estimation provides insights into the biological processes and its quantitative properties.

The parameters of biological processes are often estimated using frequentist and Bayesian approaches [4, 5]. Frequentist approaches usually exploit optimization methods to determine the maximum likelihood estimate and its uncertainty, e.g., using bootstrapping or profile likelihoods [6–8]. Bayesian approaches often rely on the sampling of the parameter posterior distribution using MCMC algorithms [9–11]. Both, optimization and sampling, are challenging for a wide range of applications encountered in computational systems biology [5, 12]. Likelihoods and posterior distributions are frequently multi-modal and possess pronounced tails (see, e.g., [4, 5]), and many applications problems possess structural and practical non-identifiabilities (see, e.g., [13–16] and references therein). This is, among others, due to scares, noise-corrupted experimental data and a features of the underlying dynamical systems, such as bistability [17, 18], oscillation [19–21] and chaos [22–24].

For optimization, a large collections of benchmark problems were established to facilitate a fair comparison of methods (see, e.g. [25]). Furthermore, optimization toolboxes are available and provide access to a large number of different optimization schemes [26, 27]. The availability of both, benchmark problems and toolboxes, is more problematic for sampling methods. To the best of our knowledge, there is no collection of benchmarking problems for sampling methods featuring dynamical systems. For MATLAB, which is frequently used for dynamical modeling in systems biology, a selection of single-chain methods is implemented in the DRAM toolbox [28]. Standard implementations for state-of-the-art multi-chain methods do however not seem to be publicly available.

In this manuscript, we address the aforementioned needs by (i) providing generic MATLAB implementations for several MCMC algorithms and (ii) compiling a collection of benchmark problems. Our code provides implementations and interfaces to several single- and multi-chain methods, including *Adaptive - Metropolis* [29–32], *Delayed Rejection Adaptive Metropolis* [28], *Parallel Tempering* [32–35], *Parallel Hierarchical Sampling* [36] and *Metropolis - adjusted Langevin algorithm* [37] with or without a preceding *multi-start optimization* [12]. Furthermore, different initialization and adaptation schemes are provided. The sampling methods are evaluated on a collection of benchmark problems – implementation provided in the Additional file 1 – featuring dynamical systems with different properties such as periodic attractors, bistability, saddle-node, Hopf and period-doubling bifurcations as well as chaotic parameter regimes and non-identifiabilities. The benchmark problems possess posterior distributions with different properties i.e., uni- and multi-modal, heavy tails and non-linear dependency structures of parameters. This collection of features which are commonly encountered in systems biology facilitates the evaluation of the sampling methods under realistic, challenging conditions. To ensure realism of the evaluations, knowledge about the posterior distribution, which is not available in practice, is not employed for selection, adaptation or tuning of methods.

To ensure a rigorous and efficient evaluation of sampling methods, we developed a semi-automatic analysis pipeline. This enabled us to evaluate >16,000 MCMC runs covering a wide spectrum of sampling methods and benchmarks. This comprehensive assessment required roughly 300,000 CPU hours. The study among others revealed the importance of using multi-chain methods and appropriate adaptation schemes. In addition, our results for the benchmark problems indicated a strong dependence of the sampling performance on the properties of the underlying dynamical systems.

## Methods

In this section, we introduce parameter estimation, sampling methods along with initialization and adaptation schemes. In addition, the analysis pipeline and the performance evaluation criteria are described.

### Mechanistic modelling and parameter estimation

We focus on ordinary differential equation (ODE) models for the mechanistic description of biological processes. ODE models are used to study a variety of biological processes, including gene regulation, signal transduction and pharmacokinetics [11, 38]. Mathematically, ODE models can be defined as

1 $$ \dot{\boldsymbol{x}}=\boldsymbol{f}(\boldsymbol{x},t,\boldsymbol{\eta}),\qquad \boldsymbol{x}(t_{0})=\boldsymbol{x_{0}}(\boldsymbol{\eta})  $$

with time *t*∈[*t*_0_,*t*_max_], state vector $\boldsymbol {x}(t)\in \mathbb {R}^{n_{x}}$ and a parameter vector $\boldsymbol {\eta }\in \mathbb {R}^{n_{\eta }}$. The vector field ***f***(***x***,*t*,***η***) and the initial conditions ***x***_***0***_(***η***) define the temporal evolution of the state variables as functions of ***η***. For biological processes, experimental limitations usually prevent the direct measurement of the state vector ***x***(*t*). Instead, measurements provide information about the observable vector ***y***(*t*). The observables depend on the state of the process, ***y***=***h***(***x***,*t*,***η***), in which ***h*** denotes the output map $\mathbb {R}^{n_{x}}\times \mathbb {R}\times \mathbb {R}^{n_{\eta }}\rightarrow \mathbb {R}^{n_{y}}$. An exemplification of ***f***(***x***,*t*,***η***) can be found in “Benchmark problems” section for each of the benchmark problems.

The measurement of the observables ***y*** yields noise corrupted experimental data $\mathcal {D} = \{(t_{k},\tilde {y}_{k})\}_{k=1}^{n_{t}}$. In the following, we assume independent, additive normally distributed measurement noise

2 $$\tilde{y}_{ik} = y_{i}(t_{k}) + \epsilon_{ik}, \qquad \epsilon_{ik}\sim\mathcal{N}\left(0,\sigma_{i}^{2}\right)  $$

in which ***σ*** denotes the standard deviation of the measurement noise and with *i*=1,…,*n*_*y*_. An example for noisy measurement data is discussed and visualized in the “Results”, “Application of sampling methods to mRNA transcription model” subsection.

The standard deviations ***σ*** are usually unknown and part of the parameter vector, i.e., ***θ***=(***η***,***σ***). The likelihood of observing the data $\mathcal {D}$ given the parameters ***θ*** is

3 $$ p(\mathcal{D}|\boldsymbol{\theta})=\prod_{i=1}^{n_{y}}\prod_{k=1}^{n_{t}}\frac{1}{\sigma_{i}\sqrt{2\pi}}\exp\left(-\frac{(\tilde{y}_{ik}-y_{i}(t_{k}))^{2}}{2\sigma_{i}^{2}}\right),   $$

in which ***y***(*t*_*k*_) depends implicitly on ***η***.

In Bayesian parameter estimation the posterior

4 $$ p(\boldsymbol{\theta}|\mathcal{D}) = \frac{p(\mathcal{D}|\boldsymbol{\theta}) p(\boldsymbol{\theta})}{p(\mathcal{D})}  $$

is considered, in which *p*(***θ***) denotes the prior and $p(\mathcal {D})$ denotes the marginal probability (being a normalization constant).

### Sampling methods

The posterior $p(\boldsymbol {\theta }|\mathcal {D})$ encodes the available information about the parameters ***θ*** given the experimental data $\mathcal {D}$ and the prior information *p*(***θ***) [39]. Accordingly, it also encodes information about parameter and prediction uncertainties. This information can be assessed by sampling from $p(\boldsymbol {\theta }|\mathcal {D})$ using MCMC algorithms.

A well-known MCMC algorithm is the Metropolis-Hastings (MH) algorithm [40, 41]. The MH algorithm samples from the posterior via a weighted random walk. Parameter candidates are drawn from a proposal distribution and accepted or rejected based on the ratio of the posterior at the parameter candidate and the current parameter. The choice of the proposal distribution is a design parameter. In practice the distribution is frequently chosen to be symmetric, e.g., a normal distribution, and centered at the current point.

**Fig. 1:**
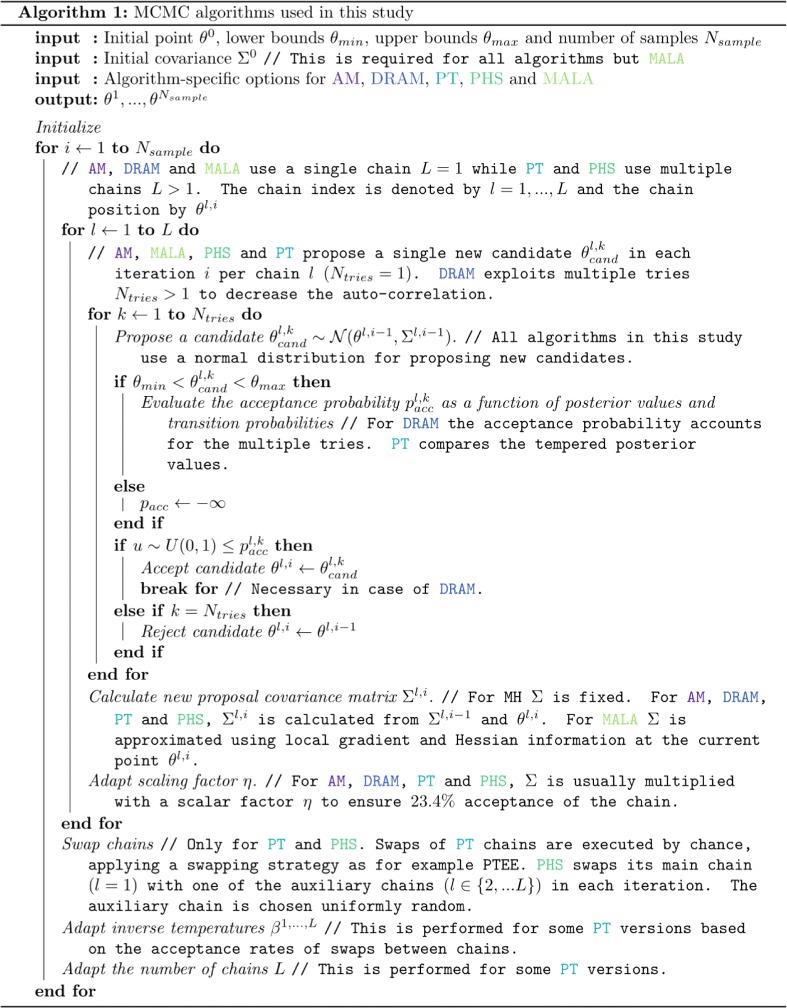
Pseudo-code for the MCMC methods used in this study. The pseudo-code highlights differences between MCMC methods using comments indicated by “ //” and the *color-coded* name of the relevant algorithm either AM, DRAM, PT, PHS or MALA

The MH algorithm has several shortcomings, including the need for manual tuning of the proposal covariance and high autocorrelation [39]. Accordingly, a large number of extensions have been developed. In the following, we introduce the three single-chain and the two multi-chain methods employed in this study. Figure 1 highlights the differences between the sampling methods employed in this study using a pseudo-code representation.

**Adaptive Metropolis (AM):** The AM algorithm is an extension of the standard MH algorithm. Instead of using a fixed proposal distribution which is tuned manually, the distribution is updated based on the already available samples. In particular, for posteriors with high correlation, this improves sampling efficiency by aligning the proposal with the posterior distribution [31]. In addition to the correlation structure, the scale of the proposal is also adapted. A commonly applied scaling scheme is based on the dimension of the problem [28, 29] while other possible schemes are based on the chain acceptance rate [34]. These scaling schemes are in the following indicated by ‘dim’ and ‘acc’, respectively.

**Delayed Rejection Adaptive Metropolis (DRAM):** To further decrease the in-chain auto-correlation, the AM algorithm has been combined with a delayed rejection method, yielding the DRAM algorithm [28]. When a candidate parameter is rejected, the algorithm tries to find a new point using the information about the rejected point. This is repeated multiple times until a certain number of tries is reached or a point is accepted. We employ the implementation provided in [28]. This implementation is exclusively based on the previously mentioned ‘dim’ adaption scheme.

**Metropolis-adjusted Langevin Algorithm (MALA):** Both AM and DRAM work best if the local and the global shape of the posterior are similar. Otherwise, the performance of the algorithm suffers, i.e. the in-chain auto-correlation increases. To circumvent this problem, the MALA makes use of the gradient, $\nabla _{\theta } p(\boldsymbol {\theta }|\mathcal {D})$, and Fisher Information Matrix [37] of the estimation problem at the current point in parameter space. This information is used to construct a proposal which is adapted to the local posterior shape [37, 42]. Gradient and Fisher Information Matrix can be computed using forward sensitivity equations [43].

**Parallel Tempering (PT):** All of the algorithms, AM, DRAM and MALA, discussed so far are single-chain algorithms which exploit local posterior properties to tune their global movement. This can make transitions between different posterior modes unlikely if they are separated by areas of low probability density. To address the issue, PT algorithms have been introduced. These algorithm sample from multiple tempered versions of the posterior $p(\mathcal {D} | \boldsymbol {\theta })^{\frac {1}{\beta _{l}}} p(\boldsymbol {\theta })$, *β*_*l*_≥1, *l*=1,…,*L*, at the same time [33–35]. The tempered posteriors are flattened out in comparison to the posterior, rendering transitions between posterior modes more likely. Allowing the tempered chains to exchange their position by chance enables the untempered chain, which samples from the posterior, to ‘jump’. For this study, we have implemented the PT algorithm as formulated by Lacki et al. [32] using AM with ‘acc’ adaption scheme or MALA for each tempered chain.

We considered different initial numbers *L*_0_ of tempered chains, adaptive *L*≤*L*_0_ or fixed numbers *L*=*L*_0_ and two different swapping strategies [32]:

- Swaps between all adjacent chains (aa)
- Swaps of chains with equal energy (ee)

are employed.

**Parallel Hierarchical Sampling (PHS):** An alternative to PT is PHS, which employs several chains sampling from the posterior [36]. Similar to PT, the idea is to start multiple auxiliary chains at different points in parameter space and to swap the main chain with a randomly picked one in each iteration. The main differences between PT and PHS are that all chains of PHS are sampling from the same distribution and that a swap between main and auxiliary chains is always accepted in PHS. The use of multiple chains can improve the mixing as different chains can employ different proposal distributions [5]. Here we apply AM(‘acc’) for each of the auxiliary chains.

### Initialization

**Fig. 2:**
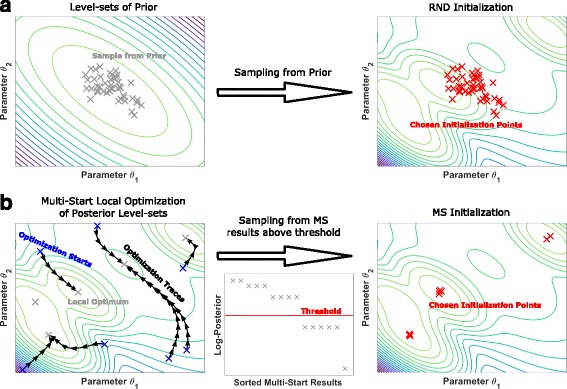
Graphical representation of initialization schemes. **a** Drawn from the prior distribution. **b** Drawn from the best results of a multi-start local optimization

The performance of sampling methods can depend on their initialization [39]. Here we consider two alternative initialization schemes: Initialization using samples from the prior distribution; and initialization using multi-start local optimization results. The methods are illustrated in Fig. 2.

**Sampling From Prior Distribution (RND):** In many applications, sampling is initialized with parameters drawn from the prior distribution. As the prior distributions are often available in closed-form, this is usually straightforward and computationally inexpensive.

**Multi-start Local Optimization (MS):** Sampling from the prior distribution frequently yields starting points with low posterior probability. Sampling methods started at these points can require a large number of iterations to reach a parameter regime with high posterior probabilities. To address this problem, initialization using multi-start local optimization has been proposed [5]. The results of multi-start local optimization provide a map of the local optima of the posterior distribution where the frequency of occurrence of a local optimum corresponds to the size of their basin of attraction. Single-chain methods are initialized at the local optima with the highest posterior probability. For multi-chain methods, we first filter the optimization results based on the difference to the best optimization result. From the remaining results initial conditions are sampled for each of the individual chains (please refer to the Additional file 1: Section 1 for further details of the initializations).

### Run repetitions

We benchmark five state-of-the-art sampling approaches for multiple settings of tuning parameters in challenging, yet low dimensional benchmark problems. In the following, these combinations – of which we consider 23 – are denoted as *scenarios*. To obtain reliable evaluation results, we perform 100 runs for each scenario thus performing 2300 runs per benchmark problem (details about the benchmark problems can be found below). Each run comprises 10^6^ iterations of a single- or multiple chains depending on the used algorithm.

### Analysis pipeline

**Fig. 3:**
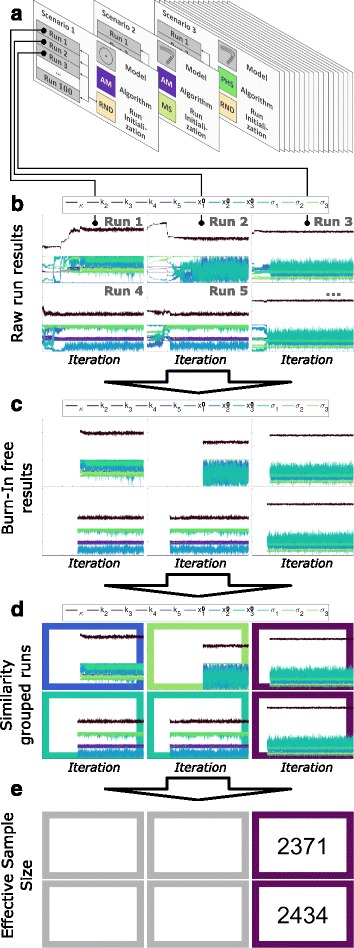
Analysis pipeline for the quantitative comparison of sampling methods. **a** Multiple MCMC runs per scenario. **b** Diversity of raw results. **c** Removal of burn-in. **d** Similarity grouping of runs across scenarios of the same benchmark problem. Each *frame color* belongs to similar chains. **e** Identification of groups with good exploration quality by comparing all groups

The sampling results for all benchmark problems and sampling strategies are analyzed using a combination of four measures: burn-in time, global exploration quality, effective sample size and computation time demand in seconds. The analysis pipeline is illustrated in Fig. 3. The pipeline exploits a combination of heuristics and statistical tests. General details are covered in the following while some further details regarding the statistical tests and heuristics can be found in Additional file 1: Sections 2 and 3.

**Fig. 4:**
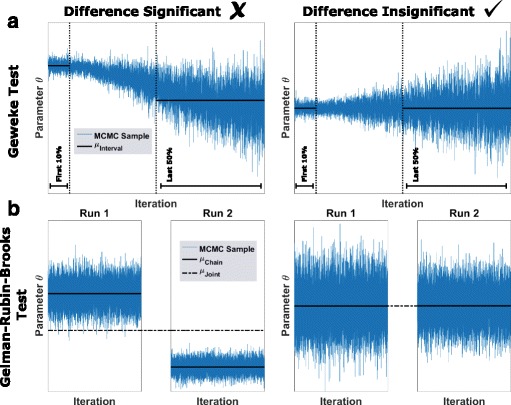
Visual representation of statistical chain diagnosis. **a** Geweke test. **b** Gelman-Rubin-Brooks test

**Burn-In (BI):** Often the first part of a Markov chain is strongly influenced by the starting point and, for adaptive methods, by the initial choice of the adaptation parameters [42]. While these effects will vanish asymptotically, for finite chain lengths there might be a large effect. To reduce these effects, the burn-in phase, in which the statistical sample mean changes substantially, is often discarded [44]. We denote the last of those iterations as *n*_*BI*_ and only the shortened chains with iteration numbers *n*_*BI*_+1 to 10^6^ are considered for further analysis. The BI is typically estimated by a visual check and validated using the Geweke test [45], which is described below and illustrated in Fig. 4 a. To circumvent a manual visual inspection, we developed an automatic approach for burn-in calculation using a sequence of Geweke tests taking Bonferroni-Holm adaptation [46] into account (see Additional file 1: Section 2 for further details).

**Exploration Quality (EQ):** An important quality measure for an MCMC algorithm is the fraction of runs which provide a representative sample from the posterior distribution for a given finite number of iterations. We denote this fraction as *EQ*.

While all MCMC algorithms considered in this manuscript converge asymptotically under mild conditions, for a finite number of samples, individual modes or tails of the posterior might be underrepresented in the chain. This problem is often adressed with statistical tests as Geweke [45] and the Gelman-Rubin-Brooks diagnostic [47]. While the Geweke test considers differences in the means of two signals (usually the beginning and the end of a MCMC chain), the Gelman-Rubin-Brooks diagnostic focuses on within-chain and between-chain variance comparison (see Fig. 4 b for a visual representation). The convergence diagnostics consider selected summary statistics, mostly the sample means, and might miss differences which are easy to spot (see, e.g. the accepted cases in Fig. 4 (right panel) and the Additional file 1: Sections 2–3 for further details about the tests). Unfortunately, convergence diagnostics provide only necessary conditions for convergence and do not necessarily reveal problems. In particular for multi-modal posterior distributions, MCMC methods sampling only from one mode pass simple convergence tests [39]. For this reason, the assessment of chain convergence is still an active field of research.

In this manuscript, we determine the EQ by first grouping individual MCMC runs of the same benchmark problem and then identify groups with members which explored the relevant parameter space well. The inspection of groups replaces the inspection of individual chains, resulting in improved efficiency and decrease of subjective judgment regarding chain convergence. The grouping is based on a pairwise distance measure between chains using the afore-described multivariate Gelman-Rubin-Brooks and Geweke diagnostics [45, 47]. If both tests are passed, the corresponding runs are assumed to be similar. Each time two runs are similar they form a group. If one of the members of a group is classified as similar to a run not yet included in the group the latter run is assigned to the entire group as well. For further details we refer to the Additional file 1: Section 3.

We compare 100 runs per scenario across algorithms (and tunings) thus evaluating 2300 runs per benchmark problem. Groups smaller than 115(5*%*) runs are neglected from further analysis. For each of the remaining groups we assess whether the posterior is explored by the group members by comparing the groups with each other. Therefore, we evaluate for each group if (i) all regions of high posterior probability and (ii) tails, found in the other groups, have been covered. In this way, we can tell if a group is not covering relevant parameter regimes found by others. This facilitates the selection of the group(s) with the best exploration properties (across algorithms). However, it can still not be ensured that the chains within the best exploring group have indeed explored the entire relevant parameter space properly.

**Effective Sample Size (ESS):** For the groups with well exploring members we compute the ESS [37, 42, 48]. The ESS accounts for the in-chain autocorrelation and is an important measure for the quality of the posterior approximation for individual chains. As the ESS is overestimated if chains sample only from individual modes of the posterior distribution, we only considered chains assigned to groups which explore the posterior well. For these chains, autocorrelation for individual parameters *θ*_*i*_ is determined using Sokal’s adaptive truncated periodogram estimator [28, 49] which is implemented in the DRAM toolbox [28]. As this is a univariate measure, we take the maximum of the autocorrelation across all *θ*_*i*_ to determine the ESS and to thin the chain.

**Computation Time:** The different sampling methods demand different computational cost. MALA requires gradient information while multi-chain methods require multiple evaluations of the (tempered) posterior probability in each iteration. To account for these differences, we evaluate the ESS per central processing unit (CPU) second, which provides a comparable measure for computational efficiency. Furthermore, we consider the efficiency reduction caused by runs which lack proper exploration. Therefore, we multiply the ESS/s value of each run with the *EQ* of the scenario. This normalization is chosen because bad runs are sometimes much faster in execution than well behaving runs, e.g. a run only proposing parameter values outside the parameter bounds is extremely swift since neither cost function nor gradients are calculated.

### Benchmark problems

**Fig. 5:**
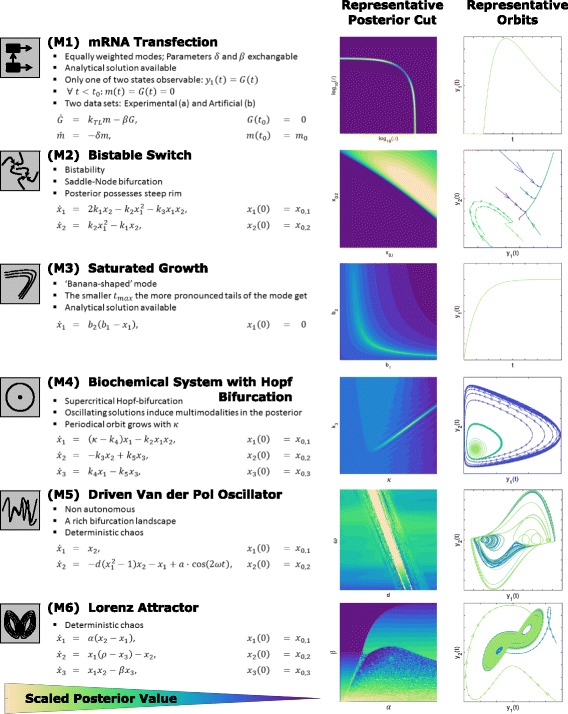
Visual summary of benchmark problems. *Left* ODE model and its properties, e.g. bifurcations. *Right* Illustration of system dynamics using posterior cuts and orbits

For the evaluation of the sampling algorithms, we established six benchmark problems for ODE constrained parameter estimation. Each benchmark problem is related to a biologically motivated ODE model. The estimation problems considered are low dimensional, yet the ODE models possess properties such as structural non-identifiabilities, bifurcations, limit-cycle oscillations and chaotic behavior. This yields posterior distributions with pronounced tails, multi-modalities and rims which makes them difficult to sample. These are common scenarios for many application problems in systems biology [4, 5, 13–24] which are difficult to identify prior to the parameter estimation. A visual summary of the benchmark problems is depicted within Fig. 5 and described in the following.

**(M1) mRNA Transfection:** This model describes the transfection of cells with GFP mRNA, its translation and degradation [50]. The observable is the protein concentration. The posterior of the estimation problem is bimodal as the exchange of the degradation rates of mRNA and protein results in the same dynamics. This ODE model is studied for experimental data (M1a) and for artificial data (M1b).

**(M2) Bistable Switch:** This model describes a bistable switch [51], a frequent motif in gene regulation [52], neuronal science [53] and population dynamics [54]. Depending on the initial condition, for given parameters, the state orbit converges to one of two steady states. This leads to a steep rim in the posterior. In addition, (M2) possesses a saddle-node bifurcation resulting in the absence of the steep rim in certain parameter regimes.

**(M3) Saturated Growth:** This model describes the growth of a population in an environment with limited resources. It is widely used to model population dynamics, i.e. immigration-death processes [55], and a variety of extensions are available. Already for the simplest model, the parameters are strongly correlated and the posterior distribution possesses ‘banana’ shaped tails if the measurement is stopped before the steady state is reached [56]. This effect can be enhanced by decreasing the maximum measurement time *t*_*max*_ when creating data.

**(M4) Biochemical System With Hopf Bifurcation:** This model describes a simple biochemical reaction network [57] with a supercritical Hopf bifurcation [58–60] as found in many biological applications [54, 61, 62]. Depending on the parameter values, the orbit of the system approaches a stable limit cycle or a stable fixed point. The posterior distribution for this problem is multi-modal but most of the probability mass is contained in the main mode.

**(M5) Driven Van Der Pol Oscillator:** This model is an extension of the Van der Pol oscillator by an oscillating input [63–66]. The input causes deterministic chaos by creating a strange attractor. Chaotic behavior can be observed in biological applications e.g. in cardiovascular models with driving pacemaker compartment [61, 67]. The posterior distribution possesses a large number of modes of different sizes and masses. This effect can be increased by creating data with larger *t*_*max*_. For chaotic systems sampling is known to be very challenging [68].

**(M6) Lorenz Attractor:** The Lorenz attractor provides an idealized description of a hydrodynamic process and can be interpreted as chemical reaction network [69]. Similar to (M5), this system is chaotic and thus possesses a multi-modal posterior distribution. However, its topology strongly differs from the one of (M5) and the chaotic behavior does not arise from a driving term.

### Priors & data generation

We consider benchmark settings with measured data (M1a) or simulated data (M1b-M6). The simulated data is obtained by simulating the models for the parameters ***θ***_*true*_ (Table 1) and adding normally distributed measurement noise. The prior distributions are uniform in the interval ***θ***∈[***θ***_*min*_,***θ***_*max*_] and the data is created using an ODE solution at ***θ***_*true*_, absolute, normally distributed noise and equidistantly spaced points in time. Information about observables is provided in Fig. 5.

**Table 1:** An overview on which priors were used and on how the data was created

	***θ***	***θ***_*min*_	***θ***_*max*_	***θ***_*true*_
(M1a)	log10(*t*_0_)	−2	1	-
	log10(*k*_*TL*_ *m*_0_)	−5	5	-
	log10(*β*)	−5	5	-
*n*_*t*_=150	log10(*δ*)	−5	5	-
*t*∈ [ 2,27]	log10(*σ*)	−2	2	-
(M1b)	log10(*t*_0_)	−2	1	log10(2)
	log10(*k*_*TL*_ *m*_0_)	−5	5	log10(5)
	log10(*β*)	−5	5	log10(0.8)
*n*_*t*_=51	log10(*δ*)	−5	5	log10(0.2)
*t*∈ [ 0,10]	log10(*σ*)	−2	2	−1
(M2)	*k*_1_	2	20	8
	*k*_2_	0	5	1
	*k*_3_	0	5	1
	*k*_4_	0	5	1
	*x*_0,1_	−3	3	2
	*x*_0,2_	−3	3	0.25
*n*_*t*_=101	$\sigma _{1}^{0}$	10^−3^	1	0.3
*t*∈ [ 0,200]	$\sigma _{2}^{0}$	10^−3^	1	0.3
(M3)	*b*_1_	0	5	1
*n*_*t*_=101	*b*_2_	0	5	0.2
*t*∈ [ 0,2.5]	*σ*_1_	10^−3^	10^2^	0.03
(M4)	*κ*	1	5	3.8
	*k*_2_	0.8	1.2	1
	*k*_3_	0.8	1.2	1
	*k*_4_	0.8	1.2	1
	*k*_5_	0.8	1.2	1
	*x*_0,1_	0	2	1
	*x*_0,2_	0	2	1
	*x*_0,3_	0	2	1
	*σ*_1_	10^−2^	2	0.75
*n*_*t*_=101	*σ*_2_	10^−2^	2	0.32
*t*∈ [ 0,200]	*σ*_3_	10^−2^	2	0.46
(M5)	*a*	2	8	5
	*d*	2	8	5
	*ω*	2	8	2.464
	*x*_0,1_	−1	3	0
	*x*_0,2_	−1	3	0
	*x*_0,3_	−1	3	1
	*σ*_1_	10^−2^	2	0.2
*n*_*t*_=101	*σ*_2_	10^−2^	2	0.8
*t*∈ [ 0,200]	*σ*_3_	10^−2^	2	0.2
(M6)	*α*	0	20	10
	*β*	0	10	$\frac {8}{3}$
	*ρ*	10	30	28
	*x*_0,1_	0	35	26.61
	*x*_0,2_	−10	10	−2.74
	*x*_0,3_	−5	5	0.95
	*σ*_1_	10^−4^	10^2^	1
*n*_*t*_=101	*σ*_2_	10^−4^	10^2^	1
*t*∈ [ 0,200]	*σ*_3_	10^−4^	10^2^	1

### Implementation

We implemented the sampling algorithms and the benchmark problems in the Parameter EStimation TOolbox (PESTO)(please refer to the “Availability of data and materials” section for a GitHub reference). This implementation in provided in Additional file 2. PESTO comes with a detailed documentation of all functionalities and the respective methods. For numerical simulation and sensitivity calculation we employed the Advanced MATLAB Interface for CVODES and IDAS (AMICI) [7, 70]. Both toolboxes are developed and available via GitHub and we provide the code used for this study in Additional file 2. The entire code basis could be transfered to other programming languages similar to MATLAB, such as Python, Octave or Julia, without major changes. A re-implementation of the tool in R would also be conceptually possible and allow for the comparison with other packages, e.g. [71].

## Results

In the following, we present the properties and the performance of sampling methods for an application problem as well as for the proposed benchmark problems.

### Application of sampling methods to mRNA transcription model

**Fig. 6:**
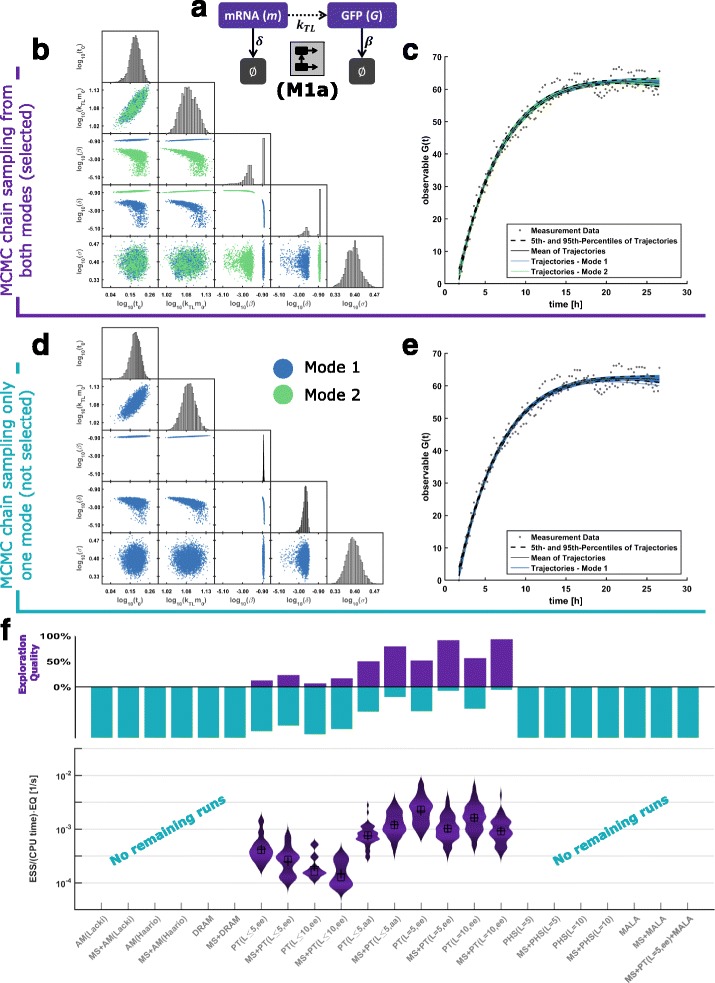
Results from benchmark problem (M1a). **a** Sketch of the translation process. **b** A bivariate scatter plot of a chain which explored both modes. **c** The corresponding trajectories of the sampled parameter points of both modes. **d** A representative chain which was not able to cover both modes. **e** The corresponding trajectories of the sample of one mode. **f** Effective sample sizes of chains which explored both modes. For several methods, no chain explored both modes, implying an effective sample size of zero

To illustrate the behavior and the properties of the different sampling methods, we consider the process of mRNA transcription ((M1), Fig. 6 a). This process has been modeled and experimentally assessed by Leonhardt et al. [50]. The ODE model possesses two state variables and five parameters. Structural analysis using the MATLAB toolbox GenSSI [15] indicated one structural non-identifiability but did not reveal its nature. Leonhardt et al. [50] derived the analytical solution of the ODE model and showed that the parameters *β* and *δ* can be interchanged without altering the output *y*. This implied that the parameters are locally but not globally structurally identifiable, giving rise to a bimodal posterior distribution (Fig. 6 b, c). As the analytical solution is in general not available, we disregard the information about the interchangeability of *β* and *δ* for the initial assessment.

We sampled the posterior distribution using several single- and multi-chain methods as well as settings and initialization schemes. The analysis of the sampling results revealed that many methods fail to sample from both modes of the posterior within 10^6^ iterations (see Fig. 6d, e). Accordingly, the exploration quality of many methods is low (Fig. 6f). We expected that the single-chain methods, AM, DRAM and MALA, always sample close to the starting point, which was indeed the case. Interestingly, we found that PHS often succeeded in moving its chain between both modes but failed to explore the modes tails properly. Merely PT, either MS or RND initialized, captured both modes in most runs (Fig. 6f). Thus, in (M1a) the conditional ESS – the ESS for the chains sampling both modes and the tails – was the highest for PT.

For most ODE constrained parameter estimation problems, information about the identifiability properties of parameters will not be available prior to the sampling. This is unfortunate as the sampling performance of all methods could be improved by exploiting such additional information. Models with parameter interchangeabilities such as (M1) are well studied in the context of mixture models. Tailored methods for such problems include post-processing methods or a random permutation sampler [72, 73]. For this simple ODE model, we evaluated the benefit of applying a post-processing strategy and found that having access to information about number and location of the posterior modes improved the sampling performance significantly for all sampling methods. (see Additional file 1: Section 4).

This application example highlights challenges arising from missing information about parameter identifiability and limitations of available sampling methods. Some of these limitations were not encountered in the manuscripts introducing the methods (e.g. [32] or [36]) as the study focused on different aspects or considered well-suited problems. The analysis of (M1a) demonstrates that even simple linear ODE models can give rise to posterior landscapes that are difficult to sample. This motivates the analysis of other (small-scale) benchmark problems.

### Benchmarking of algorithms using simulated data

To facilitate a comprehensive evaluation of sampling methods, we considered the aforementioned benchmark problems (M1-6). These benchmark problems possess a wide range of different properties regarding the underlying dynamical system (e.g. mono- and bistable) as well as the posterior distribution (e.g. unimodal/multi-modal or with/without pronounced tails). This renders the collection presented suitable for the in-depth evaluation and will facilitate the derivation of guidelines for the a priori selection of the appropriate sampling scheme.

We sampled the posterior distributions of all benchmark problems using the algorithms introduced in the “Methods” section. Different tuning parameters and initialization schemes were employed to study their influence on the sampling efficiency. For each benchmark problem we performed 100 independent runs with 10^6^ iterations. The large amount of sampling results was analyzed using the analysis pipeline illustrated in Fig. 3. The results for the individual problems (EQ and ESS) and some information about the memory usage of the different algorithms are provided in the Additional file 1: Figures S2, S4–S9.

#### Influence of posterior properties on sampling performance

**Fig. 7:**
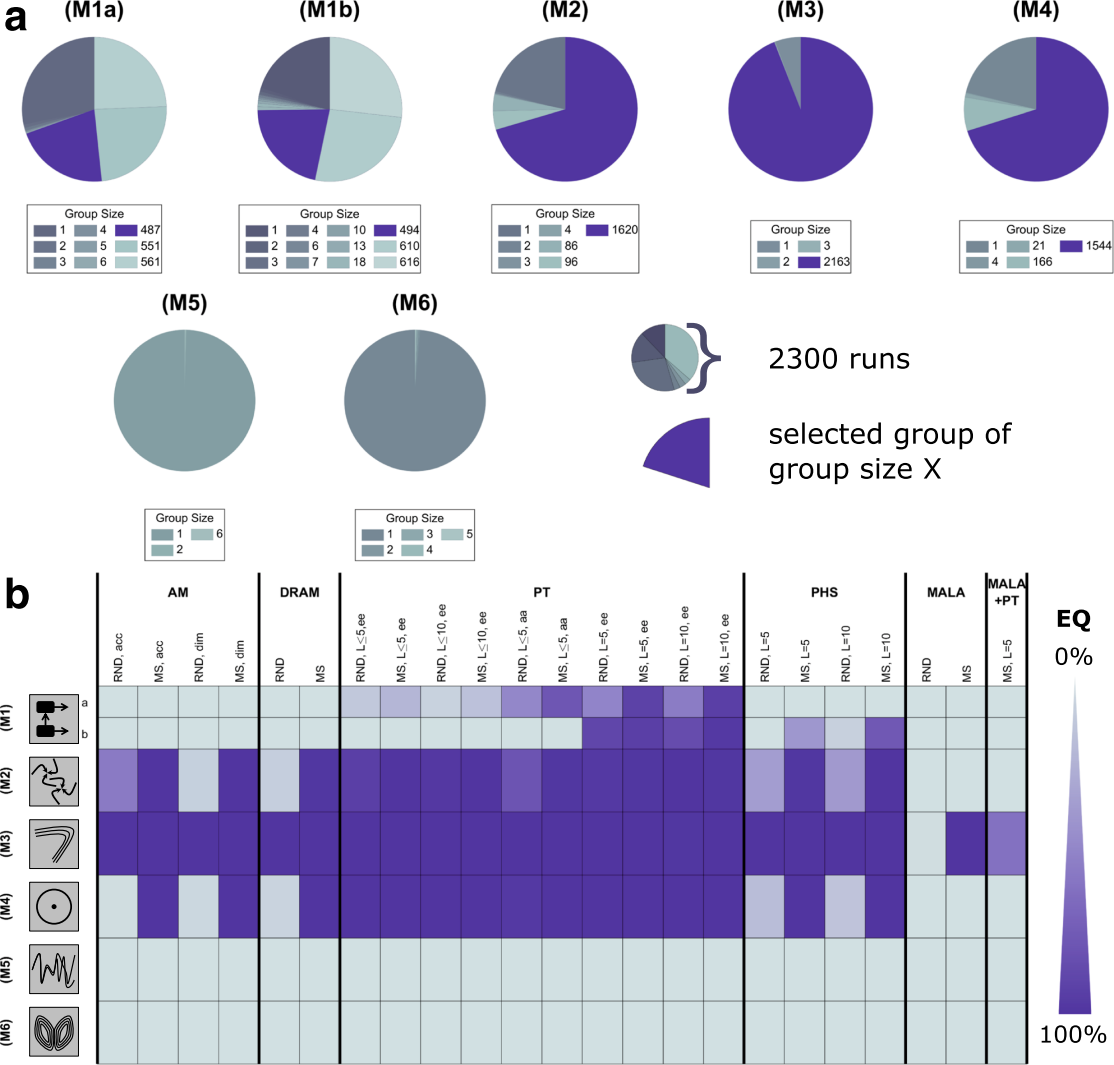
Overview of observed exploration qualities. **a** Distribution of group sizes regarding chain similarity. All groups with the same groups sizes are *colored* identically. The *coloring* scheme is indicated *below* the individual plots. **b** Exploration quality by benchmark problem (*row*) and algorithm (*column*). Each *colored square* is based on the fraction of 100 runs which were able to explore well

Given the sampling results, we asked the question how EQ depends on the benchmark problem and its properties. We found that the size of the groups of runs identified by the analysis pipeline (Fig. 7a) and the EQ (Fig. 7b) varies strongly between the benchmark problems. For problems with uni-modal (M2-3) and weakly multi-modal (M4) posteriors, the average EQ of the sampling methods was higher than 50%. For the problems with bimodal posteriors (M1a,b), 79% of the runs sampled from one of the modes and failed to explore the posterior, while 21% of the chains sampled from both modes and achieved a good exploration. For posteriors with strong multi-modalities (M5-6), all chains appear to be different and no large groups can be identified (Fig. 4a).

In terms of the dynamical properties of the underlying dynamical system, our results for the benchmark problems indicated that state-of-the-art sampling methods work well with multiple steady states and saddle-node bifurcations, as well as Hopf bifurcations and (limit cycle) oscillations resulting in weak multi-modality of the posterior, oscillating trajectories. However, these methods still fail in case of (aperiodic) oscillations/chaotic behavior and local non-identifiability resulting in strong multi-modality of the posterior.

The analysis on the level of sampling methods revealed that for (M2-4) most algorithms worked appropriately (Fig. 7b) while for (M5-6) all algorithms fail. For (M1), we observed a benefit for using PT and PHS. Since the EQ directly impacts the ESS, these observations hold true for the ESS per CPU second (Fig. 6f and Additional file 1: Figures S2–S8). Indeed, we found a strong correlation of exploration quality and sampling efficiency and identified it as the major limiting performance factor for (M1a,b) and (M5-6).

#### Comparison of single- and multi-chain methods

**Fig. 8:**
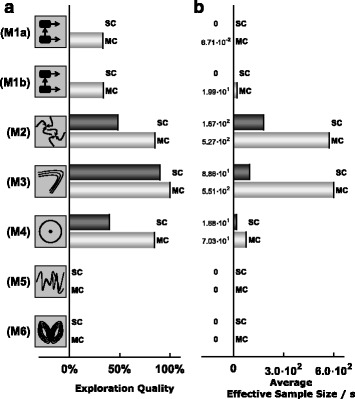
Benchmark problem wise comparison of single- and multi-chain based sampling methods. **a** EQ and **b** ESS per second computed by averaging across scenarios using single- or multi-chain sampling methods

Following the analysis of the differences between benchmark problems, we compared single- and multi-chain methods. The average performance characteristics for single- and multi-chain methods were computed by averaging over sampling methods, initialization schemes and tuning parameter choices (Fig. 8). We found that for all considered benchmark problems, multi-chain methods achieved better EQs than single-chain methods (Fig. 8a). Indeed, for several problems, multi-chain methods provided representative samples from the posterior distributions while single-chain methods sampled only individual modes. Interestingly, the improved mixing of multi-chain methods outweighed the higher computational complexity even for benchmark problems with one mode. As a result, multi-chain methods produced higher effective samples sizes and were overall computationally more efficient (Fig. 8b).

#### Comparison of initialization schemes

**Fig. 9:**
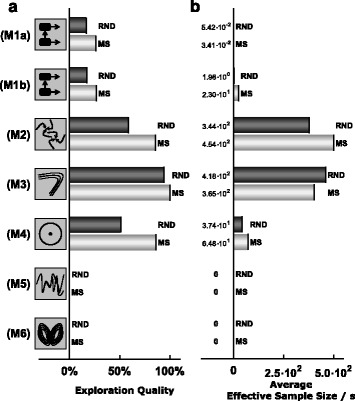
Benchmark problem wise comparison of initialization using samples from the prior (RND) and multi-start local optimization results (MS). **a** EQ and **b** ESS per second computed by averaging across scenarios using RND or MS initialization

In addition to characteristics of methods, we assessed the importance of initialization schemes. Therefore, the average performance characteristics for RND and MS initialization were computed by averaging over sampling methods and tuning parameter choices (Fig. 9). This revealed that multi-start local optimization substantially improved the EQ (Fig. 9a). The difference in the sampling efficiency (conditioned ESS per CPU second) was less pronounced than for the EQ as multi-start local optimization required additional computation time (Fig. 9b).

A detailed analysis revealed that some methods were more sensitive to the initialization than others. The performance of PT appeared to be almost independent of the initialization scheme (Fig. 7b), making it a robust choice. PHS required initialization using multi-start optimization results to achieve good EQ (Fig. 7b). Indeed, PHS initialized using samples from the prior performed poorly while PHS initialized using multi-start optimization outperformed the other methods in some cases.

#### Selection of tuning parameters and algorithm settings

To provide guidelines regarding tuning parameters and adaptation mechanisms, we carried out a fine-grained analysis of sampling method and subclasses of them. The assessment of single-chain samplers revealed that the adaptive Metropolis methods with acceptance rate dependent proposal scaling (AM(acc)) outperformed methods with dimension-dependent proposal scaling (AM(dim) and DRAM(dim)) as shown in Fig. 6f and the Additional file 1: Figures S2–S8. Delayed rejection implemented in DRAM could not compensate for the improved proposal scaling implemented in AM(acc). Furthermore, for the benchmark problems considered here, AM(acc) outperformed MALA. While AM(acc) worked for the benchmark problems with mono-modal posterior distributions, AM(dim), DRAM and MALA mostly failed to explore the posterior distribution (see Figs. 6f, 7b and Additional file1: Figures S2–S8).

The PT algorithms employed in this study used temperature and proposal density adaptation. We evaluated different swapping strategies and strategies to select the number of temperatures. The best performance characteristics were achieved with a large, fixed number of temperatures (see Fig. 6f and Additional file 1: Figures S2–S8). If few temperatures or an adaptive reduction of the number of temperatures are used, the methods are more likely to sample from a single mode. This indicates that the available methods for the reduction of the number of temperatures [32] — which worked for a series of simple examples — is not sufficiently robust. In contrast, the parallel tempering algorithms appeared to be robust with respect to the swapping strategy, with equi-energy (ee) swaps yielding superior performance.

To conclude, this section illustrated practical problems of sampling algorithms and we performed a comprehensive evaluation of sampling algorithms, initialization schemes and tuning parameters. The comprehensive evaluation provided information for the problem-specific selection of sampling strategies and beneficial combinations of settings, e.g. to combine adaptive Metropolis Parallel Hierarchical Sampling with multi-start local optimization.

## Discussion

The quantitative and qualitative properties of biological models depend on the values of their parameters. These parameters values are usually inferred using optimization or sampling methods. For optimization schemes comprehensive benchmarking results are available [12, 25, 74, 75]. In this work we complemented these results and benchmarked a selection of sampling methods.

We studied a collection of small-sized benchmark problems for ODE constrained parameter estimation with oscillating, bifurcating and chaotic solutions as well as multi-stable steady states and non-identifiabilities. These model properties lead to pronounced tails, multiple modes and rims in the posterior distributions. Some of these challenges can be addressed by employing additional information about the model and tools like structural identifiability analysis (see “Application of sampling methods to mRNA transcription model” section). However, in applications, it might not be possible to avoid non-identifiabilities, e.g., if the biological interpretation needs to be conserved or prediction uncertainties need to be quantified. By considering benchmark problems with a diverse set of features, this study provided an unbiased comparison for available sampling methods.

As a by-product of our presented benchmarking study we considered the effect of properties of the ODE model, such as Hopf-bifurcation and multi-stability, onto the performance of sampling algorithms. As most models of biological systems are nonlinear, high-dimensional and possess multiple positive and negative feedback loops [76], a single model can usually exhibit different properties in different parameter regimes. As the biologically relevant regimes in parameter spaces are usually unknown prior to the parameter estimation, knowledge about the dynamic properties cannot be employed and the use of robust sampling methods is beneficial. We previously expected bifurcations to strongly impact the sampling efficiency. This, however, was not the case. Instead, we observed that chaotic regimes have a strong influence on the sampling efficiency and might even render it intractable. This is consistent with previous finding and expected as “chaotic likelihood functions, while ultimately smooth, have such complicated small scale structure” [68].

To derive guidelines for sampling method selection, we assessed a range of single- and multi-chain samplers. This revealed that most state-of-the-art sampling methods require a large number of iterations to provide a representative sample from multi-modal posterior distributions even in low-dimensional parameter spaces. Multi-chain methods clearly outperformed single-chain methods, as reported earlier (see, e.g., [5, 21] and references therein), even for unimodal posterior distributions. The reliability and performance of all sampling methods except PT was substantially improved when initialized using optimization results instead of samples from the prior. Interestingly, for the benchmarks considered in this manuscript, PT performed better without novel adaptation schemes for the number of temperatures [32]. This is in contrast to results for posterior distributions in the original publication [32] – for which we achieved the same results using our implementation –, suggesting that additional research is required. Furthermore, this emphasizes the importance of realistic test problems. The comparison of dimension-dependent proposal scaling [28] and acceptance-rate-dependent proposal scaling [34], which was to the best of our knowledge not published before, revealed the superiority of the latter. From this insight a range of single- and multi-chain methods can benefit. Overall, PHS with optimization-based initialization performed best for uni-modal posterior landscapes while PT performed most robustly regarding all posteriors.

Beyond the evaluation of algorithms, the results demonstrate the importance of performing multiple independent runs of sampling methods starting from different points in parameter space [5]. Most algorithms provide merely a representative sample in a fraction of the runs. In addition to standard sampling diagnostics (e.g. convergence tests like Gelman-Rubin-Brooks [45]), our extended analysis pipeline takes into account the EQ while minimizing the need for subjective visual inspection. Our results confirm the need to evaluate sampling methods by not only taking into account the ESS of the generated runs but the overall EQ as important measure for algorithmic robustness.

The benchmark problems considered in this study are low-dimensional but resemble essential features of parameter estimation problems in systems biology. While the precise quantitative results might depend on the selection of the benchmarks, the qualitative findings should be transferable. To verify this, a range of application problems should be considered. Furthermore, while several classes of sampling methods have been considered, the study of additional methods would be beneficial. In particular the assessment of Hamiltonian Monte Carlo (HMC) based algorithms such as NUTS or Wormhole Monte Carlo [77, 78], region-based methods [79], Metropolis-in-Gibbs methods [80], Transitional MCMC [81], sequential Monte Carlo methods [82] or additional proposal adaptation strategies [71] would be valuable. For ODE models for which the full conditional distribution of the parameters can be derived, also Gibbs samplers might be used [83]. Furthermore, a comparison with non-sampling-based approximation methods, e.g. variational methods [84] or approximation methods [85] could be interesting.

## Conclusion

In summary, our comprehensive evaluation revealed that even state-of-the-art MCMC algorithms have problems to sample efficiently from many posterior distributions arising in systems biology. Problems arose in particular in the presence of non-identifiabilities and chaotic regimes. The examples provided in manuscripts presenting new algorithms are often not representative and a more thorough assessment on benchmark collections should be required (as is common practice in other fields). The presented study provides a basis for future developments of such benchmark collections allowing for a rigorous assessment of novel sampling algorithms. In this study, we already used six benchmark problems with common challenges to provide practical guidelines for the selection of sampling algorithms, adaptation and initialization schemes. Furthermore, the presented results highlight the need to address chain exploration quality by taking into account multiple MCMC runs which can be compared with each other before calculating effective sample sizes. The availability of the code will simplify the extension of the methods and the extension of the benchmark collection.

## Additional files

Additional file 1 Supplementary Notes. Covering additional details about the analysis pipeline and sampling results. (PDF 1200 kb)

Additional file 2 Supplementary Code. Containing a standalone implementation of methods, benchmark problems, data sets and analysis tools used in this study. (ZIP 6953 kb)

## Abbreviations

AM: Adaptive metropolis
aa: All adjacent
acc: Acceptance based adaption
BI: Burn-In
CPU: Central processing unit
dim: Dimension based adaption
DRAM: Delayed rejection adaptive metropolis
ee: Equal energy
ESS: Effective sample size
EQ: Exploration quality
MALA: Metropolis-adjusted Langevin algorithm
MCMC: Markov chain Monte Carlo
MH: Metropolis-hastings
MS: Multi-start local optimization
ODE: Ordinary differential equation
PT: Parallel tempering
PHS: Parallel hierarchical sampling
RND: Sampling from prior distribution
