# Supplementary material for: Comprehensive benchmarking of Markov chain Monte Carlo methods for dynamical systems
Source: BMC Syst Biol. 2017 Jun 24;11:63. doi: 10.1186/s12918-017-0433-1 (PMC5482939; doi:10.1186/s12918-017-0433-1)
Supplement: Supplementary file 1 — Supplementary Notes. Covering additional details about the analysis pipeline and sampling results. (PDF 1200 kb) [file 12918_2017_433_MOESM1_ESM.pdf]

RESEARCH

SUPPLEMENT: Comprehensive Benchmarking of Markov Chain Monte Carlo Methods for Dynamical Systems

Benjamin Ballnus[1,2], Sabine Hug[1], Kathrin Hatz[3], Linus Görlitz[3], Jan Hasenauer[1,2] and Fabian J Theis[1,2*]

1 Initialization Scheme Details

To initialize the sampling algorithms we use samples from the prior (RND initialization) or results of a multi-start local optimization run (MS initialization). An illustration of the initialization is provided in Supplement Figure S1.

RND Initialization: In this initialization scheme we draw 100 parameter points from our uniform priors. For single-chain algorithms, these points are distributed to the 100 runs per scenario. For multi-chain algorithms, we take different (but overlapping) sequences of L points from the 100 available points and distribute these onto the L chains of each run. Each scenario has a different random seed.

MS Initialization: For each run we draw 1000 parameters vectors according to a Latin Hypercube scheme and use them as initial values for a multi-start local optimization (as presented in [1]). The local optimization for each starting point is performed using the MATLAB build-in routine *fmincon* with 'interior-point'.

For single-chain methods the maximum a posteriori (MAP) estimate obtained using the multi-start local optimization is employed as initial parameter vector.

For multi-chain methods, we filter the optimization results based on the posterior difference to the MAP and only take into account the best $u \leq 1000$ optimization results $\{\boldsymbol{\theta}^{(j)}, j = 1, ..., u\}$. To determine a reasonable number u we define $R_i = 2(\log(L(\boldsymbol{y}|\boldsymbol{\theta^{(1)}})p(\boldsymbol{\theta^{(1)}})) - \log(L(\boldsymbol{y}|\boldsymbol{\theta^{(i)}})p(\boldsymbol{\theta^{(i)}})))$, $i = 1, \ldots, 1000$ where $\boldsymbol{\theta}_i$ are sorted descending by their corresponding posterior values from best to worst posterior values. Therefore, the parameter values are sorted, such that we obtain a sequence $\boldsymbol{\theta}^{(1)}$, $\boldsymbol{\theta}^{(2)}, \ldots, \boldsymbol{\theta}^{(1000)}$, such that $p(\mathcal{D}|\boldsymbol{\theta}^{(1)})p(\boldsymbol{\theta}^{(1)}) \geq p(\mathcal{D}|\boldsymbol{\theta}^{(2)})p(\boldsymbol{\theta}^{(2)}) \geq \cdots \geq p(\mathcal{D}|\boldsymbol{\theta}^{(1000)})p(\boldsymbol{\theta}^{(1000)})$. From this sequence the first u members are selected such that for all $i = 1, \ldots, u$, the log-posterior ratio:

$$R_i = 2 \log \left(\frac{p(\mathcal{D}|\boldsymbol{\theta}^{(1000)})p(\boldsymbol{\theta}^{(1000)})}{p(\mathcal{D}|\boldsymbol{\theta}^{(u)})p(\boldsymbol{\theta}^{(u)})} \right) \qquad (1)$$

is larger than the inverse χ^2 distribution with one-degree of freedom with an α-level of 0.001. This is motivated by the likelihood ratio test. For the remaining optimization results $\{\boldsymbol{\theta}^{(j)}, j = 1, \ldots, u\}$ we define weights w_j calculated with the log-posterior values such that $w_j = 1-(p(\boldsymbol{\theta}^{(1)}|\mathcal{D})-p(\boldsymbol{\theta}^{(j)}|\mathcal{D}))/(p(\boldsymbol{\theta}^{(j)}|\mathcal{D})-p(\boldsymbol{\theta}^{(u)}|\mathcal{D})) \in [0,1]$. The weight w_j is used to draw the initial values from $\boldsymbol{\theta_j}$) for our multi-chain MCMC run. This heuristic takes into account both, the height and width of the modes of the posterior distribution. The width is encoded in the frequency which a certain local optima is recovered.

2 Automated Burn-In Calculation via Sequential Geweke Test

The different sampling methods yield burn-ins of different lengths. To determine the length of the burn-in phase automatically, we employ the Geweke test multiple times. The Geweke test is originally used to compare the sample means $\bar{\mu}_{0\%-10\%}$ and $\bar{\mu}_{50\%-100\%}$ of the first 10% and the last 50% of a chain [2] while accounting for the respective spectral variance approximations $\hat{\sigma}^2_{0-10\%}$ and $\hat{\sigma}^2_{50-100\%}$ of the interval sample means. The comparison is based on the z-score:

$$z = \left| \frac{\bar{\mu}_{0\%-10\%} - \bar{\mu}_{50\%-100\%}}{\sqrt{\hat{\sigma}^2_{0\%-10\%} + \hat{\sigma}^2_{50\%-100\%}}} \right|. \qquad (2)$$

The z-score is used to test the hypothesis that the sample means are different against the null-hypothesis that the sample means $\bar{\mu}_{0\%-10\%}$ and $\bar{\mu}_{50\%-100\%}$ are equal. We consider a z-score greater than $z_0 = 2$ as significant. If the z-score is smaller than 2, the null hypothesis is not rejected, suggesting that the burn-in phase is over.

*Correspondence: fabian.theis@helmholtz-muenchen.de
[1]Helmholtz Zentrum München - German Research Center for Environmental Health, Institute of Computational Biology, Ingolstädter Landstraße 1, 85764 Neuherberg,Germany
Full list of author information is available at the end of the article

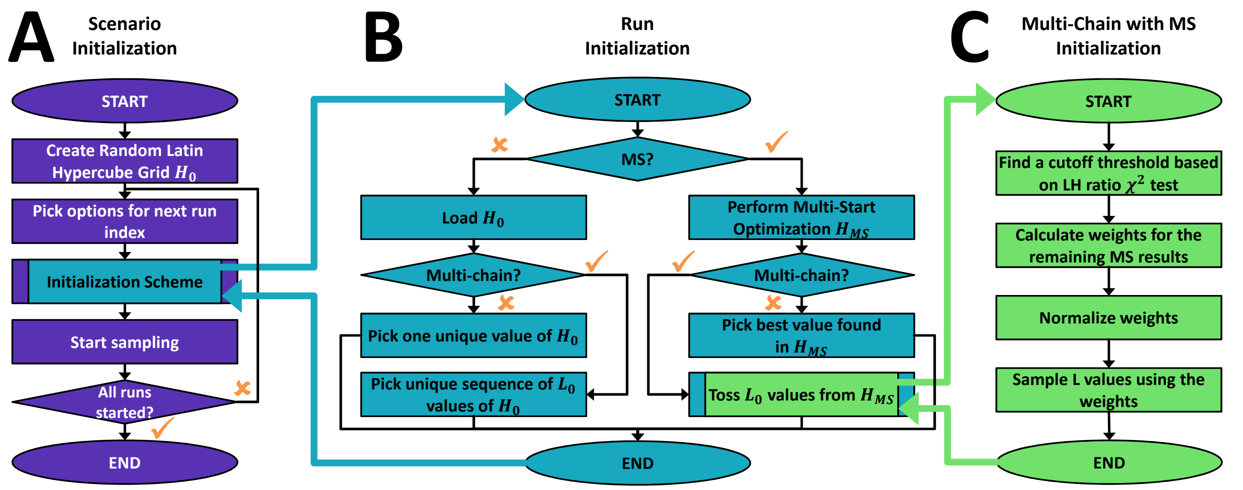

Figure S1 Sampling and initialization work flowchart. **(A)** Flowchart for sampling. **(B)** Flowchart for initialization of sampler, distinguishing single- and multi-chain methods as well as random and optimization-based initialization. **(C)** Flowchart for the selection of starting points for optimization-based initialization.

For automatic detection of the BI length, we split up the raw chain into 40 equally sized intervals. Then, we perform the Geweke test on subsets of these intervals. Initially the Geweke test is performed on the chain composed of all segments $1, ..., 40$. If the resulting z-score is too high, we test the chain composed of the second to the last segment $2, ..., 40$. If the z-score is still too high, we test the third to the last segment $3, ..., 40$ and so on until we have tested all sub-chains or the z-score falls below the threshold.

We adjust the significance level z_0 for each subsequent test with the Bonferroni-Holm [3] correction. Thus, we sort the obtained z-scores in descending order (as they are anti-proportionally related to the p-values, which should be sorted ascendingly) and normalize the corresponding z_0 of the test as $\tilde{z_0} = z_0/k$ where k is the sorted index $k \in \{1, ..., 40\}$ of the test.

Once the z-score is lower than the significance level $\tilde{z_0}/k$, we choose all iterations with $i \leq n_{BI}$ to be discarded. As all MCMC chains are multidimensional with $n_\theta > 1$ and the Geweke test is a univariate test, we always test all parameter dimensions individually and then use the worst case, i.e. the highest BI found for all dimensions.

3 Obtaining Groups of Similar MCMC Chains

To obtain groups of similar chains for the same benchmark problem in robust fashion, we compare all pairs of runs using a Gelman-Rubin-Brooks [4] and Geweke test.

The Gelman-Rubin-Brooks test compares the variance-covariance matrix W of $\boldsymbol{\theta}$ within a set of chains with the variance-covariance B of $\boldsymbol{\theta}$ between the set of chains [4]. Here $\theta_i^{(jt)}$ is the ith element of the parameter vector in chain j at iteration t. We define the variance-covariance matrix within the chains as

$$W = \frac{1}{(n-1)m} \sum_{j=1}^{m} \sum_{t=1}^{n} (\theta^{(jt)} - \bar{\theta}^{(j)})(\theta^{(jt)} - \bar{\theta}^{(j)})^T \quad (3)$$

and the variance-covariance matrix between the chains as

$$B/n = \frac{1}{m-1} \sum_{j=1}^{m} (\bar{\theta}_j - \bar{\theta})(\bar{\theta}_j - \bar{\theta})^T. \quad (4)$$

with means

$$\bar{\theta}^{(j)} = \frac{1}{n} \sum_{t=1}^{n} \theta^{(jt)} \quad \text{and} \quad \bar{\theta} = \frac{1}{nm} \sum_{j=1}^{m} \sum_{t=1}^{n} \theta^{(jt)}. \quad (5)$$

Here n is the number of iterations and m the number of chains being compared (here always $n = 2$).

Any rotationally invariant distance measure between W and B/n can be used to determine if the chains are sufficiently similar or not [4]. Brooks and Gelman proposed

$$R = \frac{n-1}{n} + \frac{m+1}{m}\lambda, \quad (6)$$

as distance measure where λ is the largest eigenvalue of $W^{-1}B/n$. This multivariate measure is an upper bound for its univariate counterpart (see [4]).

We found this similarity test to be a good choice if combined with a Geweke test *between* the chains (note, this is a different Geweke test than the one used in Supplement Section 2). This Geweke test is used on the last parts of both chains after Burn-In removal further shortening the longer chain so that both chains have equal iteration numbers remaining. The Gelman-Rubin-Brooks test is used to compare the covariances within and across chains while the Geweke test compares the chain means. The conservativeness of both approaches can be controlled by modifying the thresholds R_0 and z_0 which are used to R and z. The tests are passed, if $R < R_0$ and $z < z_0$. We set $R_0 = 1.05$ and $z_0 = 0.05$ fairly conservative to make it more likely to overestimate the number of groups to underestimate it. If both tests are passed, we consider two chains to be sufficiently similar to be assigned to the same group. Each time two runs are classified as similarly they form a group and linked, existing groups are getting merged.

4 Memory Usage

We have tested the memory usage of our implementation of the sampling methods by applying them in the largest considered benchmark problem (M4) with 10^4 iterations. We performed 10 independent runs, 5 runs with MS initialization and 5 runs with RND initialization. Figure S2 shows memory consumption due to the sampling process. To generate these results, the undocumented `profile -memory` functionality of MATLAB2016b was used on a desktop computer. Since MALA is more sensitive to bad initialization than the other methods, the RND initialized runs fail to start. Thus, in that case the computation time is very short.

5 Resolving Non-Identifiability Leads to Higher Sampling Efficiency

For (M1a), an analysis of the analytical solution for the output trajectory $y(t)$ reveals that the parameters β and δ are interchangeable. These information can be employed by constructing more efficient samples, e.g. by using a random permutation sampler, or by post-processing the results [5, 6]. In this study, we considered post-processing. Instead of applying the analysis pipeline to the raw chains, we first process the chains by switching β and δ so that $\beta > \delta$ holds. The resulting increase in EQ and ESS per second are shown in Figure S3. The sampling performance improves significantly for almost all algorithms. The single-chain algorithms benefit the most. This highlights the importance of usage of additional information for sampling problems. Unfortunately, additional information – as used here – is often not accessible in practice.

6 Detailed Results of each Benchmark Problem

Figures S4–S10 illustrate the performance of the considered sampling approaches for benchmark problems (M1a) to (M6). The exploration quality as well as the sampling efficiency (effective sample size per second) is indicated.

Author details

[1]Helmholtz Zentrum München - German Research Center for Environmental Health, Institute of Computational Biology, Ingolstädter Landstraße 1, 85764 Neuherberg,Germany. [2]Technische Universität München, Center for Mathematics, Chair of Mathematical Modeling of Biological Systems, Boltzmannstraße 15, 85748 Garching, Germany. [3]Bayer AG, Engineering & Technologies, Applied Mathematics, Kaiser-Wilhelm-Allee, 51368 Leverkusen, Germany.

References

1. Raue, A., Kreutz, C., Theis, F.J., Timmer, J.: Joining forces of Bayesian and Frequentist methodology: a study for inference in the presence of non-identifiability. Philosophical Transactions of the Royal Society A: Mathematical, Physical and Engineering Sciences **371**(1984), 20110544 (2013)
2. Brooks, S.P., Roberts, G.O.: Assessing convergence of Markov chain Monte Carlo algorithms. Statistics and Computing **8**(4), 319–335 (1998)
3. Holm, S.: A simple sequentially rejective multiple test procedure. Scandinavian Journal of Statistics **6**(2), 65–70 (1979)
4. Brooks, S.P., Gelman, A.: General methods for monitoring convergence of iterative simulations. Journal of computational and graphical statistics **7**(4), 434–455 (1998)
5. Jasra, A., Holmes, C.C., Stephens, D.A.: Markov chain Monte Carlo methods and the label switching problem in Bayesian mixture modeling. Statistical Science, 50–67 (2005)
6. Papastamoulis, P., Iliopoulos, G.: On the Convergence Rate of Random Permutation Sampler and ECR Algorithm in Missing Data Models. Methodology and Computing in Applied Probability **15**(2), 293–304 (2013)

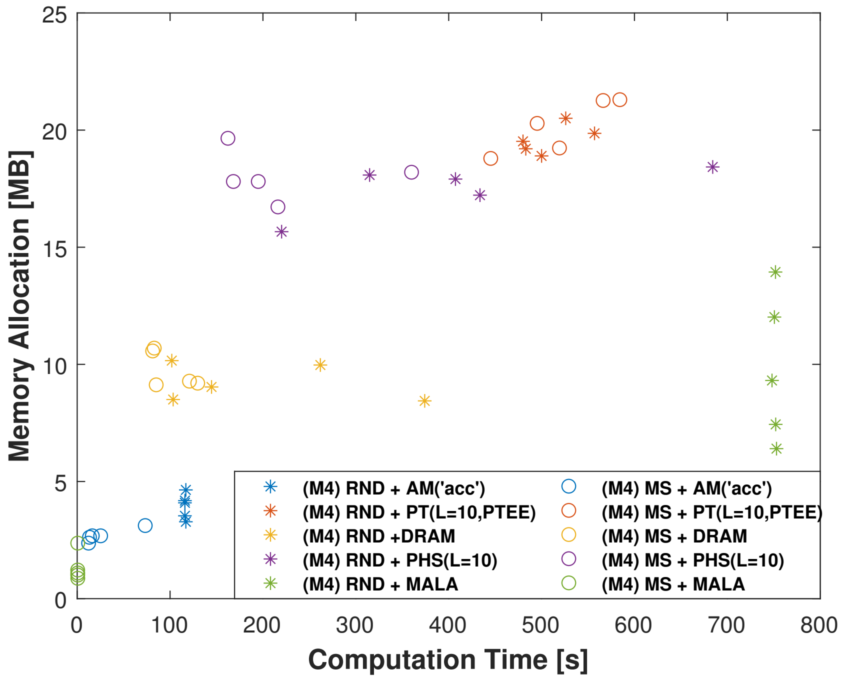

Figure S2 Memory consumption of different algorithms in (M4). Memory allocation and computation time of 5 runs with MS initialization and 5 runs with RND initialization for 10^4 iterations.

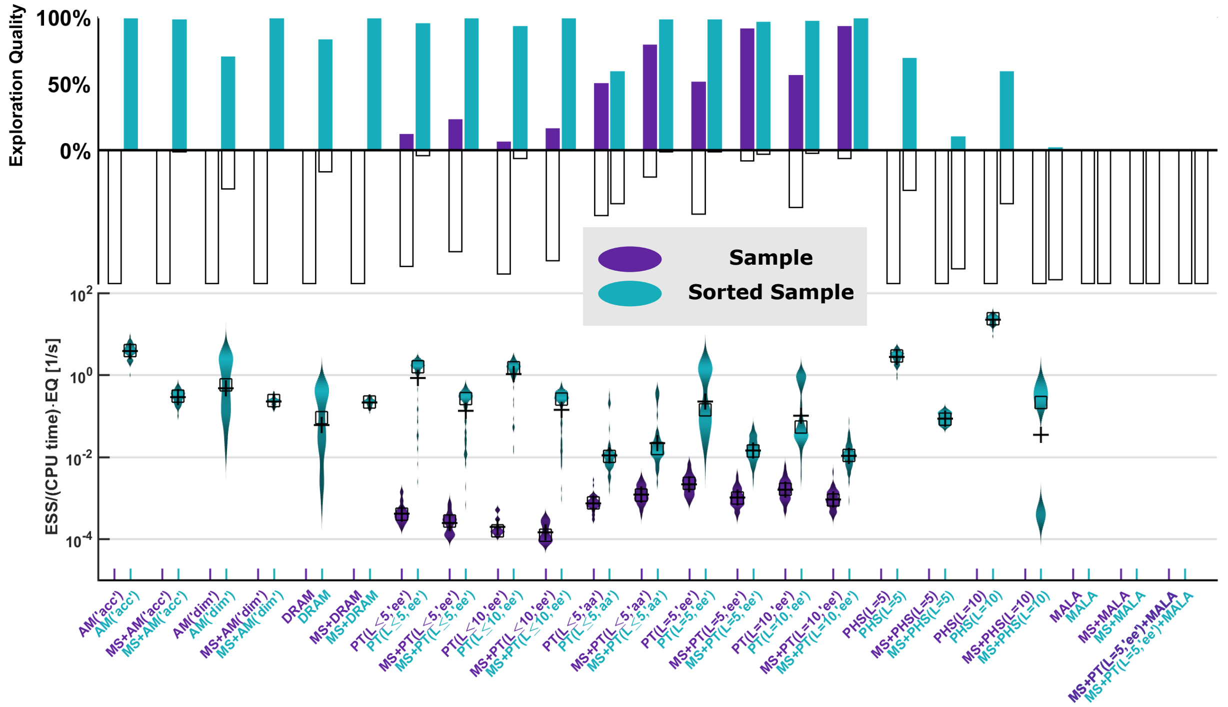

Figure S3 Sampling performance comparison for (M1a). Upper Panel: Exploration Quality. **Lower Panel:** Distribution of Effective Sample Size per second regarding runs which contribute to Exploration Quality. Results for the original sample and the post-processed (= sorted) sample are shown.

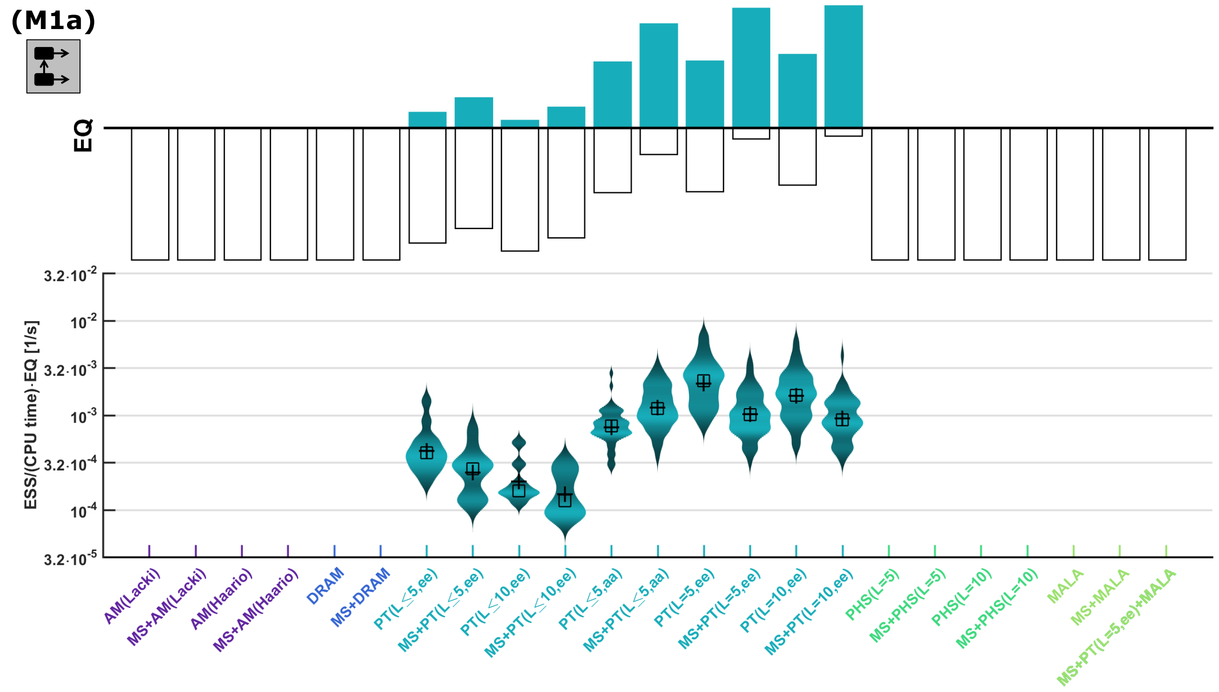

Figure S4 Exploration quality (top) and sampling efficiency (bottom) for problem (M1a). A complete white bar in the upper plot indicates that less than 5% of the runs of the respective algorithm explored the posterior distribution. In this case, the sampling efficiency is not evaluated, otherwise, the distribution of corrected ESS across well exploring runs is illustrated.

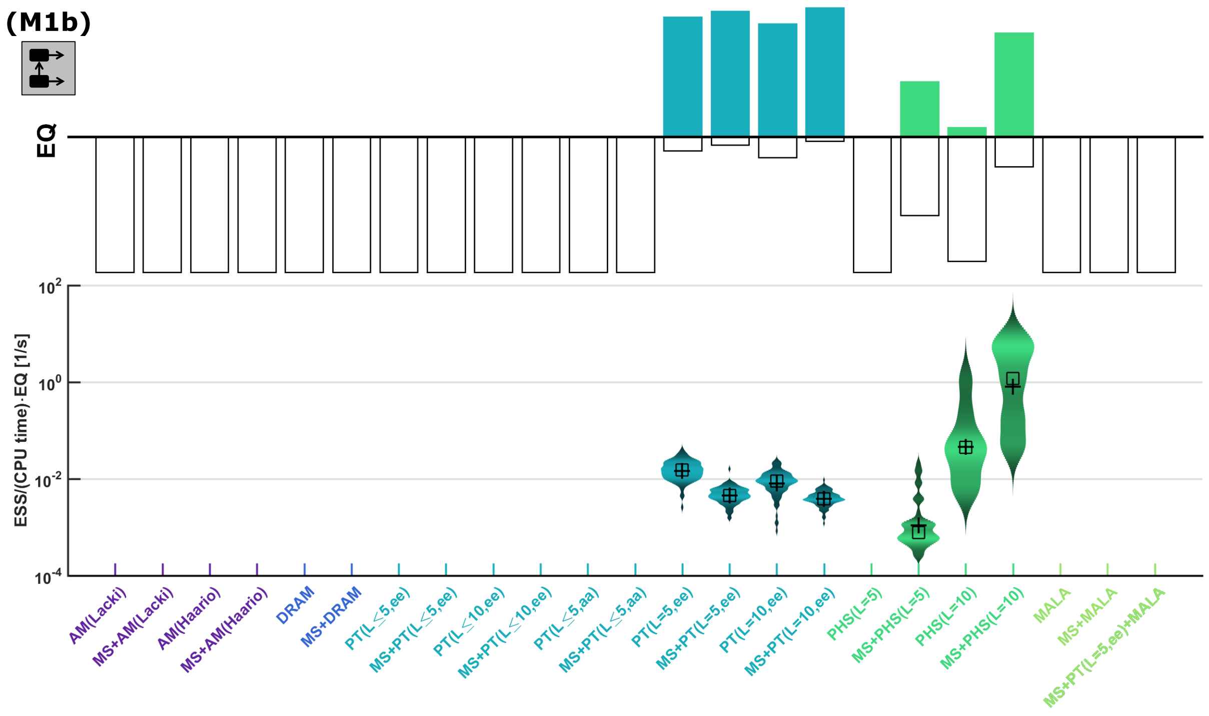

Figure S5 Exploration quality (top) and sampling efficiency (bottom) for problem (M1b). A complete white bar in the upper plot indicates that less than 5% of the runs of the respective algorithm explored the posterior distribution. In this case, the sampling efficiency is not evaluated, otherwise, the distribution of corrected ESS across well exploring runs is illustrated.

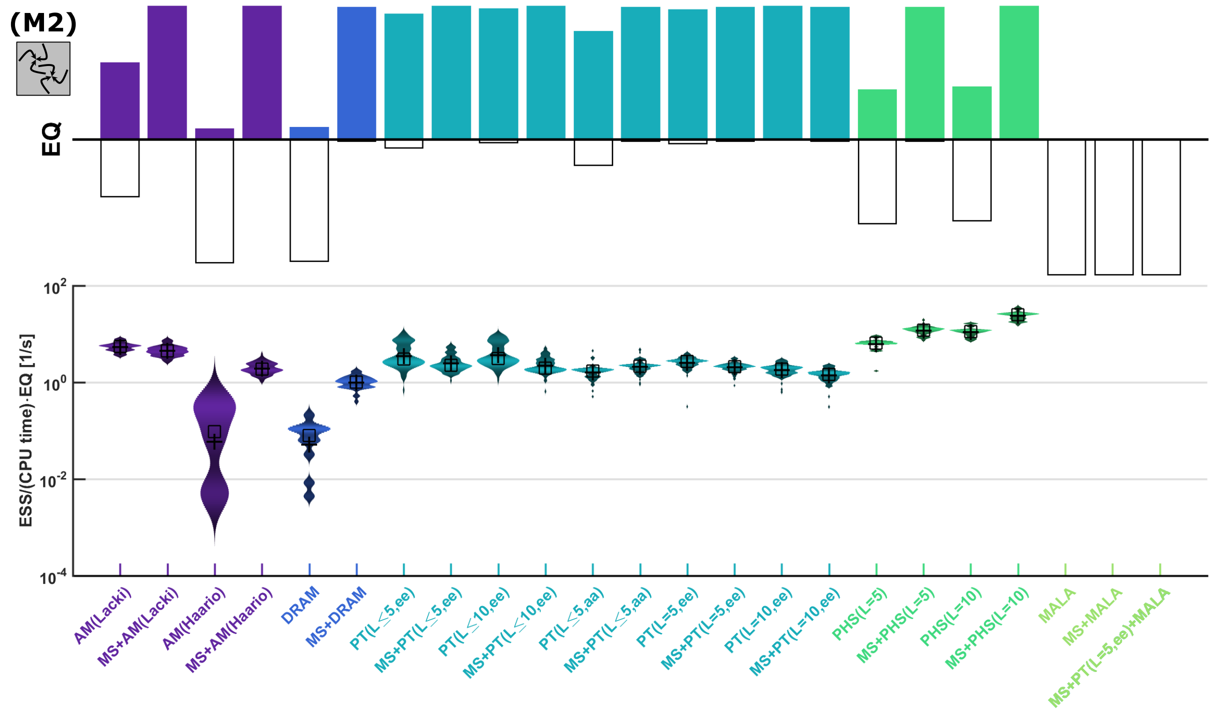

Figure S6 Exploration quality (top) and sampling efficiency (bottom) for problem (M2). A complete white bar in the upper plot indicates that less than 5% of the runs of the respective algorithm explored the posterior distribution. In this case, the sampling efficiency is not evaluated, otherwise, the distribution of corrected ESS across well exploring runs is illustrated.

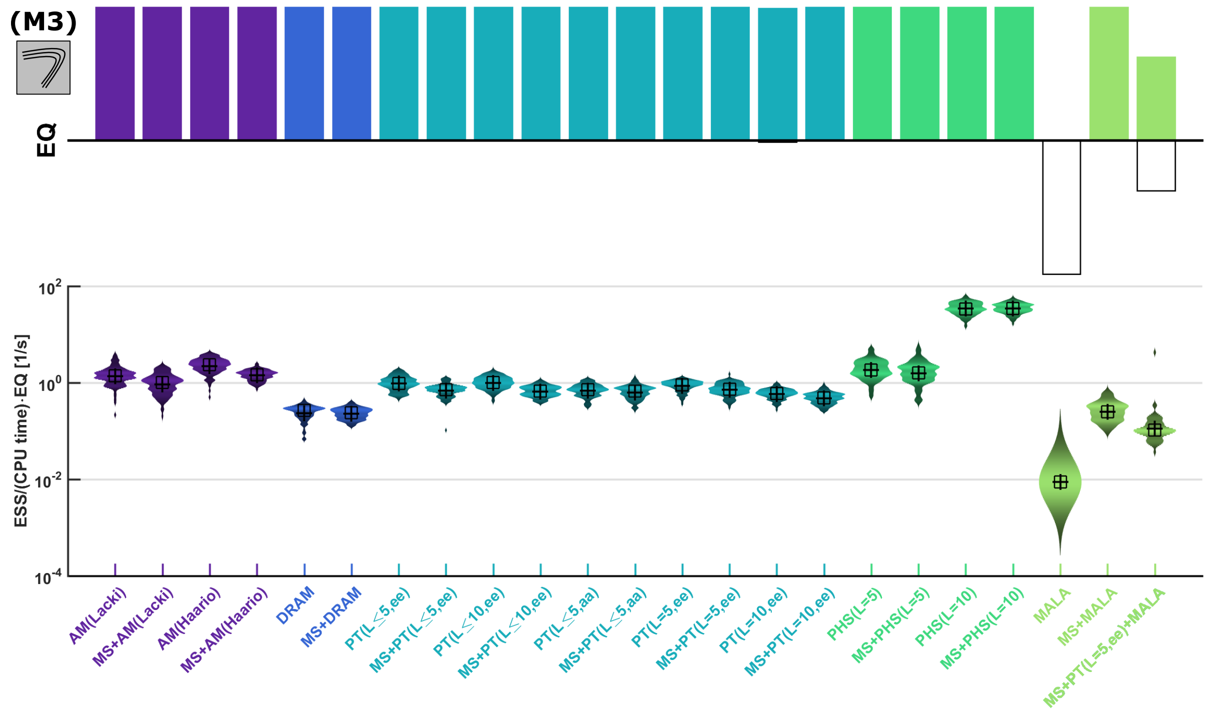

Figure S7 Exploration quality (top) and sampling efficiency (bottom) for problem (M3). A complete white bar in the upper plot indicates that less than 5% of the runs of the respective algorithm explored the posterior distribution. In this case, the sampling efficiency is not evaluated, otherwise, the distribution of corrected ESS across well exploring runs is illustrated.

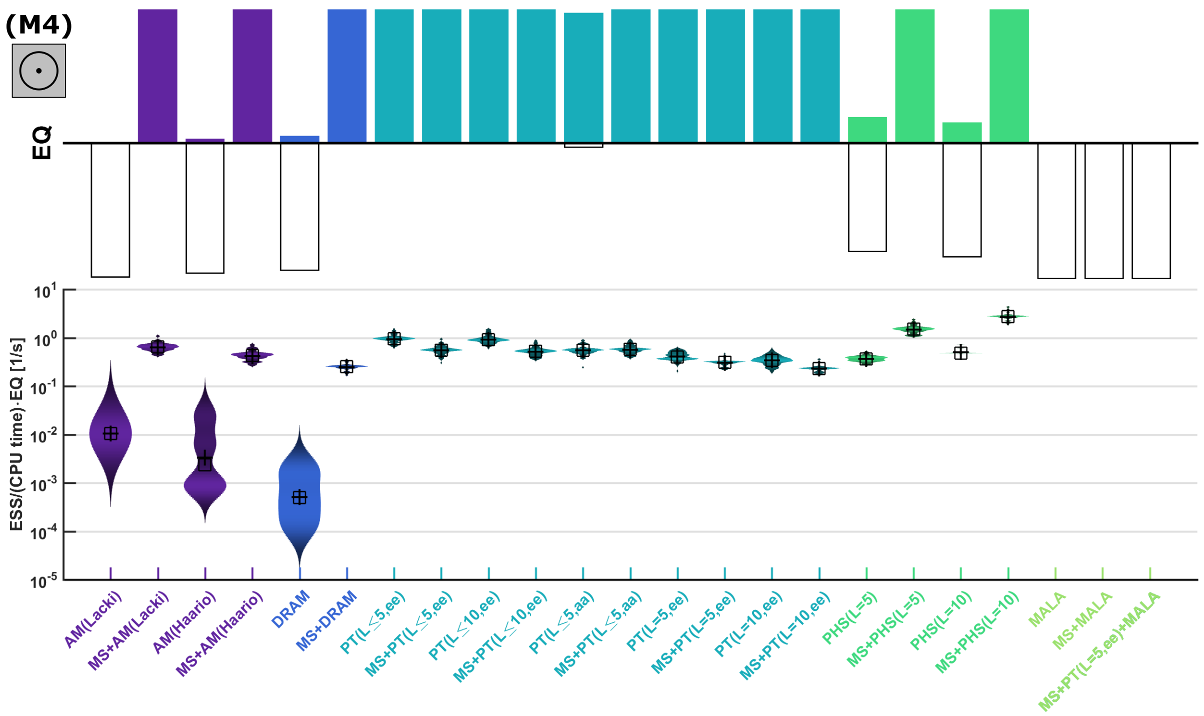

Figure S8 Exploration quality (top) and sampling efficiency (bottom) for problem (M4). A complete white bar in the upper plot indicates that less than 5% of the runs of the respective algorithm explored the posterior distribution. In this case, the sampling efficiency is not evaluated, otherwise, the distribution of corrected ESS across well exploring runs is illustrated.

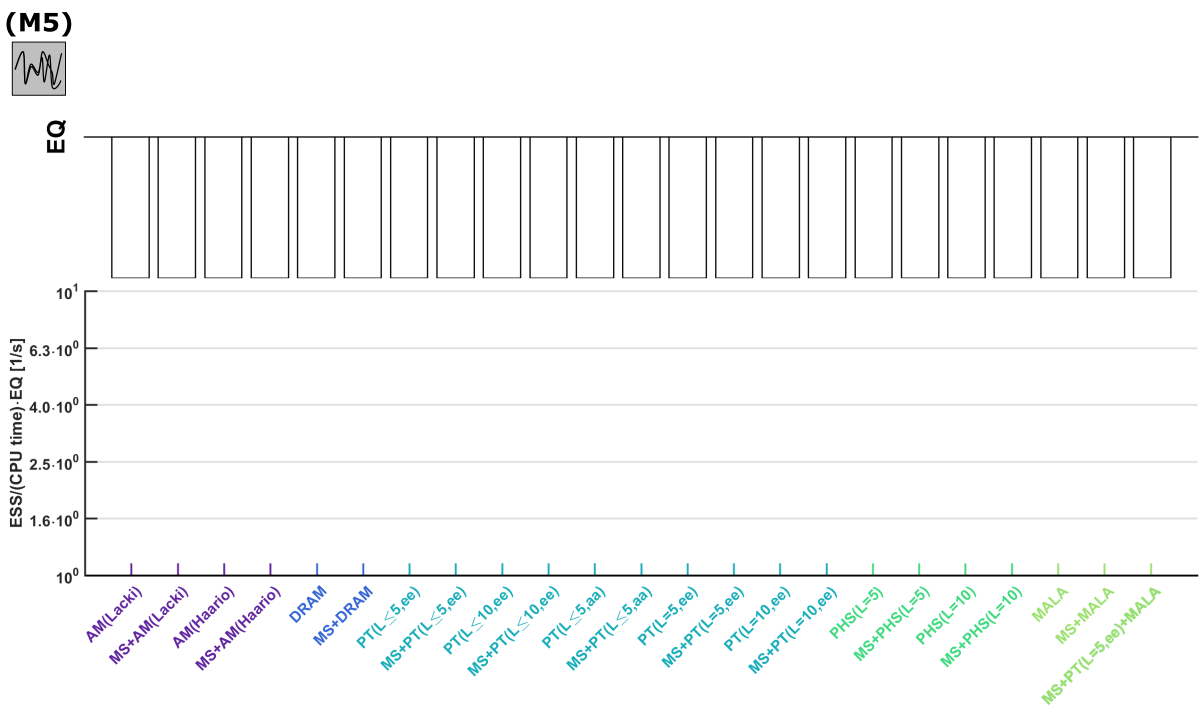

Figure S9 Exploration quality (top) and sampling efficiency (bottom) for problem (M5). A complete white bar in the upper plot indicates that less than 5% of the runs of the respective algorithm explored the posterior distribution. In this case, the sampling efficiency is not evaluated, otherwise, the distribution of corrected ESS across well exploring runs is illustrated. In this benchmark problem none of the algorithms has shown $EQ > 0$.

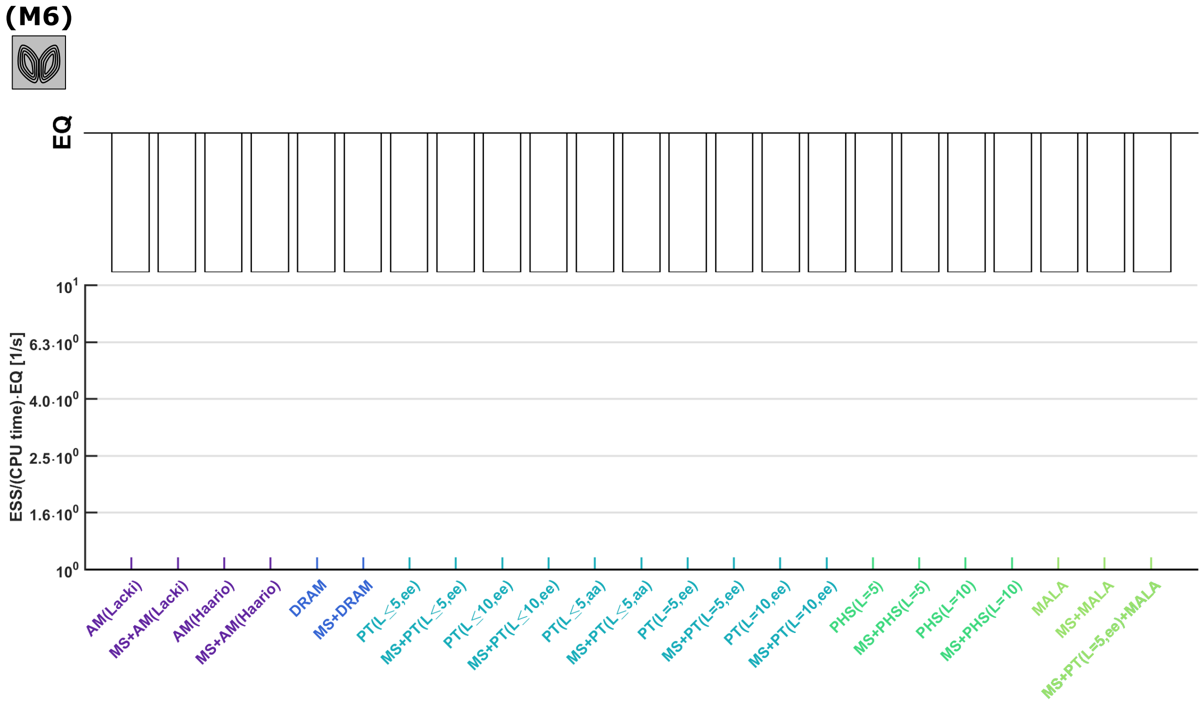

Figure S10 Exploration quality (top) and sampling efficiency (bottom) for problem (M6). A complete white bar in the upper plot indicates that less than 5% of the runs of the respective algorithm explored the posterior distribution. In this case, the sampling efficiency is not evaluated, otherwise, the distribution of corrected ESS across well exploring runs is illustrated. In this benchmark problem none of the algorithms has shown $EQ > 0$.
